# Culture-Confirmed Invasive Meningococcal Disease in Canada, 2010 to 2014: Characterization of Serogroup B *Neisseria meningitidis* Strains and Their Predicted Coverage by the 4CMenB Vaccine

**DOI:** 10.1128/mSphere.00883-19

**Published:** 2020-03-04

**Authors:** Raymond S. W. Tsang, Dennis K. S. Law, Rosita De Paola, Maria Giuliani, Maria Stella, Jianwei Zhou, Saul Deng, Giuseppe Boccadifuoco, Marzia Monica Giuliani, Laura Serino

**Affiliations:** a Vaccine Preventable Bacterial Diseases, National Microbiology Laboratory, Public Health Agency of Canada, Winnipeg, Manitoba, Canada; b GSK, Siena, Italy; University of Missouri-Kansas City School of Medicine

**Keywords:** serogroup B, MATS assay, disease, invasive, meningococcal

## Abstract

The molecular epidemiology of culture-confirmed invasive meningococcal disease (IMD) in Canada from 2010 to 2014 was studied with an emphasis on serogroup B Neisseria meningitidis (MenB) isolates, including their predicted coverage by the 4CMenB vaccine. The mean annual incidence rates of culture confirmed IMD varied from 0.19/100,000 in Ontario to 0.50/100,000 in New Brunswick and 0.59/100,000 in Quebec. In both Quebec and Atlantic region, MenB was significantly more common than other serogroups, while in other provinces, both MenB and serogroup Y (MenY) were almost equally common. The majority of MenB cases (67.0%) were in those aged ≤24 years, while most MenC (75.0%) and MenY (69.6%) cases were in adults more than 24 years old. The 349 MenB isolates were grouped into 103 sequence types (STs), 90 of which belonged to 13 clonal complexes (CCs). A large number of 4CMenB antigen genes were found among the Canadian MenB, which is predicted to encode 50 factor H binding protein (fHbp) types, 40 NHBA types, and 55 PorA genotypes. Provinces and regions were found to have their own unique MenB STs. A meningococcal antigen typing system assay predicted an overall MenB coverage by 4CMenB to be 73.6%, with higher coverage predicted for the two most common STs: 100% for ST154 and 95.9% for ST269, leading to higher coverage in both the Atlantic region and Quebec. Higher coverage (81.4%) was also found for MenB recovered from persons aged 15 to 24 years, followed by strains from infants and children ≤4 years old (75.2%) and those aged 5 to 14 years (75.0%).

**IMPORTANCE** Laboratory surveillance of invasive meningococcal disease (IMD) is important to our understanding of the evolving nature of the Neisseria meningitidis strain types causing the disease and the potential coverage of disease strains by the newly developed vaccines. This study examined the molecular epidemiology of culture-confirmed IMD cases in Canada by examining the strain types and the potential coverage of a newly licensed 4CMenB vaccine on Canadian serogroup B N. meningitidis strains. The strain types identified in different parts of Canada appeared to be unique as well as their predicted coverage by the 4CMenB vaccine. These data were compared to data obtained from previous studies done in Canada and elsewhere globally. For effective control of IMD, laboratory surveillance of this type was found to be essential and useful to understand the dynamic nature of this disease.

## Abstract (precis)

Laboratory surveillance of invasive meningococcal disease (IMD) is important to our understanding of the evolving nature of the Neisseria meningitidis strain types causing the disease and the potential coverage of disease strains by the newly developed vaccines. This study examined the molecular epidemiology of culture-confirmed IMD cases in Canada by examining the strain types and the potential coverage of a newly licensed 4CMenB vaccine on Canadian serogroup B N. meningitidis strains. The strain types identified in different parts of Canada appeared to be unique as well as their predicted coverage by the 4CMenB vaccine. These data were compared to data obtained from previous studies done in Canada and elsewhere globally. For effective control of IMD, laboratory surveillance of this type was found to be essential and useful to understand the dynamic nature of this disease.

## INTRODUCTION

Neisseria meningitidis is a Gram-negative bacterium that can be classified into twelve serogroups (A, B, C, E, H, I, K, L, W, X, Y, and Z) based on antigenic specificities present on the surface polysaccharide capsule (1). However, most diseases are caused by N. meningitidis strains that belong to six serogroups (A, B, C, W, X, and Y) (2, 3). Also, strains of N. meningitidis that are associated with invasive meningococcal disease (IMD), particularly those capable of causing outbreaks and epidemics, can be classified by multilocus sequence typing (MLST) into a few clonal complexes (CCs) that are related to sequence types (STs) with no more than three of their seven MLST loci being different (https://pubmlst.org/neisseria/) (2, 4).

One of the virulence factors of N. meningitidis is the surface polysaccharide capsule, which can shield the bacterium from complement activation and its subsequent lytic action. Plain and protein-conjugated polysaccharide capsule vaccines from serogroups A (MenA), C (MenC), W (MenW), and Y (MenY) have been developed and licensed for use globally, including Canada (5). A pentavalent capsule-based conjugate vaccine against serogroups A, C, W, X, and Y has recently been developed and is currently under early clinical trial (6).

Countries with meningococcal conjugate vaccine programs have experienced changes in the epidemiology of IMD with drastic decrease in diseases caused by serogroups included in the vaccines used (7). In Canada, in the post-MenC conjugate vaccine era, most IMD are now caused by serogroups B and Y (8). With most provinces and territories adopting some form of the quadrivalent A, C, Y, and W conjugate vaccine programs (9), further changes in the epidemiology of IMD may be expected.

Currently, there are no capsule-based vaccines to protect against IMD caused by serogroup B N. meningitidis (MenB) because MenB capsule is poorly immunogenic and may have the potential to cause autoimmunity due to the unique structure of the MenB capsule showing similarity to a human host tissue antigen (10, 11). However, two protein-based MenB vaccines have been developed and licensed for use in Canada. One licensed MenB vaccine (Trumenba) is based on two factor H binding proteins (fHbps) with one selected from each subfamily (12). The other MenB vaccine, 4CMenB (Bexsero), is made up of three recombinant meningococcal proteins (subfamily B or variant 1 fHbp peptide 1; *Neisseria* heparin binding antigen [NHBA], peptide 2, and *Neisseria* adhesion A [NadA], peptide 3.8), combined with an outer membrane vesicle (OMV) component made from the MenB strain NZ98/254 expressing the PorA P1.4, as the major antigen (13).

Previously, it has been determined that the overall predicted coverage of 4CMenB on 157 Canadian MenB isolates collected from 2006 to 2009 was 66% (95% confidence interval, 46 to 78%), and the predicted coverages for the two most common STs were 95 and 100% for ST269 and ST154, respectively (14). Since strain types may change over time, we embarked on a study using more recent isolates collected from 2010 to 2014. Our objectives included a detailed study on the molecular epidemiology of culture-confirmed IMD in Canada, with an emphasis on MenB, and to determine the predicted protective coverage of 4CMenB on a representative sample of 250 Canadian MenB isolates. Here, we discuss our results in the context of our earlier findings as well as data collected from other countries.

## RESULTS

### Culture confirmed IMD cases in Canada.

From 2010 to 2014, 550 individual N. meningitidis case isolates were received at the National Microbiology Laboratory (NML). Their serogroup distribution by year is shown in Table 1 . The average number of case isolates per year during the study period was 110, but the number varied from year to year with a high of 133 isolates in 2011 to a low of 84 isolates in 2014, a drop of 36.8% that was mainly due to fewer number of MenB isolates.

**TABLE 1:** Distribution of invasive N. meningitidis serogroups and mean annual incidence rate by provinces in Canada 2010 to 2014^*a*^

Yr and serogroup	No. of isolates received from										
	BC	AB	Central		ON	QC	Atlantic region				All
			SK	MB			NS	PEI	N&L	NB	
2010											
B	1	3	0	4	12	42	1	0	2	4	69 (58.0)
C	3	1	2	3	3	2	0	0	0	0	14 (11.8)
Y	1	4	2	1	13	3	1	0	0	1	26 (21.8)
W	2	1	0	1	1	1	0	0	0	0	6 (5.0)
O	2*	1**	0	0	0	0	0	0	0	1**	4 (3.4)
Total											119
											
2011											
B	5	4	3	0	16	50	2	0	1	3	84 (63.2)
C	0	0	0	0	1	1	0	0	0	0	2 (1.5)
Y	6	2	2	1	18	5	0	0	0	0	34 (26.0)
W	1	3	0	1	2	3	0	0	0	0	10 (7.5)
O	1**	0	0	0	1‡	1†	0	0	0	0	3 (2.3)
Total											133
											
2012											
B	9	5	0	1	18	41	0	1	0	6	81 (72.3)
C	1	6	0	0	1	3	0	0	0	0	11 (9.8)
Y	3	0	3	0	6	5	0	0	0	0	17 (15.2)
W	0	1	0	1	0	1	0	0	0	0	3 (2.7)
O	0	0	0	0	0	0	0	0	0	0	0 (0.0)
Total											112
											
2013											
B	3	3	3	4	9	47	0	0	0	2	71 (69.6)
C	2	3	0	0	1	0	0	0	0	0	6 (5.9)
Y	5	5	0	1	8	2	0	0	0	0	21 (20.6)
W	0	1	0	1	0	2	0	0	0	0	4 (3.9)
O	0	0	0	0	0	0	0	0	0	0	0 (0.0)
Total											102
											
2014											
B	5	3	0	3	6	21	2	0	2	2	44 (52.4)
C	1	1	0	0	2	4	0	0	0	0	8 (9.5)
Y	4	2	1	2	13	5	0	0	0	0	27 (32.1)
W	1	1	0	1	1	0	0	0	0	0	4 (4.8)
O	0	0	0	1‡	0	0	0	0	0	0	1 (1.2)
Total											84
											
MAR^*b*^	0.24	0.25	0.35		0.19	0.59	0.26^*c*^				0.31

a Serogroups B (serogroup B), C (serogroup C), Y (serogroup Y), W (serogroup W), O (other serogroups of N, meningitidis) are included. *, one serogroup E, one nonencapsulated; **, one serogroup E; †, one serogroup X; ‡, one serogroup Z. Region abbreviations: BC, British Colombia; AB, Alberta; SK, Saskatchewan; MB, Manitoba; ON, Ontario; QC, Quebec; NS, Nova Scotia; PEI, Prince Edward Island; N&L, Newfoundland and Labrador; NB, New Brunswick. “All” refers to all Canada (*n* = 550). The percentage for the year is indicated in parentheses.

b MAR, mean annual rate, i.e., the mean annual incidence rate per 100,000 population based on our laboratory surveillance of culture-confirmed invasive meningococcal disease case isolates.

c The mean annual incidence rate per 100,000 population in New Brunswick was 0.50.

The distribution of IMD case isolates by province showed 43.5% of the isolates were from Quebec, 24.0% were from Ontario, 10.2% were from British Columbia, 9.1% were from Alberta, 7.6% were from Saskatchewan/Manitoba, and 5.6% were from the Atlantic region (Table 1). Based on the number of isolates received from each province, the mean annual incidence rates of culture-confirmed IMD cases were determined, and they ranged from a low of 0.19/100,000 in the province of Ontario to a high of 0.59/100,000 in the province of Quebec (Table 1). Besides this difference, serogroup distribution of N. meningitidis case isolates also showed remarkable geographical variations. In the Atlantic region and Quebec, MenB was significantly more common than other serogroups combined (the *P* values were 0.001 for both Quebec and the Atlantic region), while in the rest of the country, there was no such significant difference. In both Quebec and the Atlantic region, MenB was responsible for 84.1 and 90.3% of the IMD isolates, respectively. Both MenC and MenY isolates were uncommon there. Of the 239 and 31 IMD isolates in Quebec and Atlantic region, only 4.2 and 0%, respectively, were MenC and the corresponding figures for MenY were 8.4 and 6.5%, respectively. In contrast, in Ontario both MenB and MenY were common, being responsible for 46.2 and 43.9%, respectively, of the IMD case isolates there; and MenC was present at a frequency of 6.1%. In British Columbia, Alberta, and Saskatchewan/Manitoba, the frequencies of MenB, MenC, and MenY were 41.1, 12.5, and 33.9%; 36.0, 22.0, and 26.0%; and 42.9, 11.9, and 31.0%, respectively. MenW caused between 2.7 and 7.5% of the culture-confirmed IMD cases during the study period and was not a major cause of IMD.

Of the 348 (99.7%) MenB IMD cases with age information, 38.2% occurred in those under the age of 5 years, 8.3% in children aged 5 to 14 years, 20.7% in adolescents and young adults (aged 15 to 24 years), 23.0% in adults aged 25 to 64 years, and 9.8% in those aged 65 years and above (Table 2). Of the 40 MenC IMD cases with age information, 75.0% occurred in adults over the age of 24 years and 17.5% occurred in adolescents and young adults. Similarly, MenY IMD cases occurred mainly in adults (69.6%), followed by adolescents and young adults (18.4%) and children under the age of five years (8.8%).

**TABLE 2:** Age distribution of 550 invasive meningococcal disease (IMD) cases and incidence rates by serogroup in Canada, 2010 to 2014

Age range (yr) or IR^*a*^	No. of cases by serogroup					Total
	B	C	Y	W	Other^*b*^	
Age range						
<1	65	1	7	3	0	76
1–4	68	0	4	5	0	77
5–14	29	2	4	1	0	36
15–24	72	7	23	4	4	110
25–44	29	8	18	2	0	57
45–64	51	12	35	5	1	104
≥65	34	10	34	7	3	88
NI^*c*^	1	1	0	0	0	2
Total	349	41	125	27	8	550
						
IR	0.197	0.023	0.071	0.015	0.005	0.310

a The mean annual incidence rate (IR) of culture-confirmed IMD per 100,000 population categorized by serogroup.

b That is, the other serogroup category includes four serogroup E samples, two serogroup Z samples, one serogroup X sample, and one nonencapsulated N. meningitidis sample.

c NI, no age information was available.

### Invasive MenB in Canada: clonal analysis.

Of the 349 MenB isolates, 329 were grouped into 90 STs which could be clustered into thirteen CCs (see Table S1 in the supplemental material). The remaining 20 MenB belonged to 13 STs that could not be assigned to any known CC. The five most commonly encountered MenB CCs were CC269 (48.1%) (with *n* = 168 isolates), CC41/44 (28.6%) (*n* = 100), CC213 (4.0%) (*n* = 14), CC32 (4.0%) (*n* = 14), and CC35 (3.7%) (*n* = 13); overall, 309 MenB isolates (88.5%) belonged to these CCs.

10.1128/mSphere.00883-19.1 TABLE S1 Distribution of 349 invasive serogroup B N. meningitidis case isolates in Canadian provinces* from 2010 to 2014 according to their clonal complexes (CCs) and corresponding sequence types (STs) as determined by multilocus sequence typing. Download Table S1, PDF file, 0.3 MB. © Crown copyright 2020. 2020 Crown This content is distributed under the terms of the Creative Commons Attribution 4.0 International license.

Although CC269 was the most commonly found MenB CC in Canada, its presence was most noticeable in the province of Quebec since 86.3% of the CC269 (145 of 168 isolates) were detected in that province, where 72.1% (145 of 201 isolates) of their MenB were typed as CC269, and 91.7% (133 of 145 isolates) of them belonged to a single ST, ST269. Overall, of the 168 Canadian MenB isolates in the CC269, 142 (84.5%) were typed as ST269, while the remaining 26 isolates were grouped into 13 different STs. The other region with a predominant MenB CC was in the Atlantic provinces, where 75.0% of the MenB (21 of 28 isolates) were typed as CC41/44 and 81.0% (17 isolates) belonged to ST154. In the rest of Canada, their invasive MenB were usually grouped into three or four different CCs. For example, in Ontario, 37.7, 18.0, 9.8, and 9.8% of their invasive MenB belonged to CC41/44, CC269, CC32, and CC35, respectively (Table S1). Although western Canada (British Columbia, Alberta, Saskatchewan, and Manitoba) has only 31.6% of the country’s population, 78.6% CC213 isolates were recovered from there.

Unlike the Canadian MenB CC269 which was mostly clonal, the 100 Canadian MenB CC41/44 isolates were genetically very diverse with 46 different STs found. The Simpson’s diversity index of MenB in the CC269 was 0.285, while the value for MenB in the CC41/44 was 0.909. Despite this genetic diversity of CC41/44, two STs combined (ST154 with 26 isolates and ST571 with 15 isolates) were responsible for 41.0% of the isolates in this CC. Geographical distribution of these two STs were also noticed. Of the 15 ST571 isolates, 13 (86.7%) were found in the province of Quebec, with the remaining 2 isolates found in the neighboring province of Ontario. However, in Quebec, ST571 was only responsible for 6.5% of their invasive MenB. For ST154, 17 or 65.4% of this ST were found in the Atlantic region, where it was responsible for 60.7% of their MenB IMD cases or 81.0% of their CC41/44. Most (*n* = 14) of the ST154 IMD isolates were from the province of New Brunswick. The other ST154 isolates were found in Ontario (three isolates), Saskatchewan/Manitoba (three isolates), and in British Columbia, Alberta, and Quebec (each with one isolate).

When the Simpson’s diversity index was calculated on the MenB population based on their MLST data, the MenB population in Ontario was found to have an index of 0.9858, while the index for strains in Quebec was 0.5588. The diversity index for MenB in British Columbia, Alberta, and Saskatchewan/Manitoba were 0.9723, 0.9673, and 0.9542, respectively. In the Atlantic region, the diversity index of MenB was 0.3651.

### Genetic diversity of Canadian MenB strains by DNA sequencing of the 4CMenB vaccine antigen genes.

Targeted gene sequencing of the 349 MenB strains revealed 50 fHbp peptide types in which 23 belonged to subfamily B (variant 1), and 27 belonged to subfamily A (17 were variant 2 and 10 were variant 3). There were 236 isolates (67.6%) determined to have *fhbp* genes encoding subfamily B (variant 1) peptides; 87 isolates (24.9%) were predicted to encode variant 2 peptides; and 24 isolates (6.9%) were predicted to encode variant 3 peptides. There were also two isolates (0.6%) with *fhbp* gene mutations (one with a frameshift mutation and one with an internal stop codon) leading to no peptide synthesis. The six most commonly found fHbp peptide types were peptide 4, 13, or 15 (variant 1); peptide 16 or 19 (variant 2); or peptide 45 (variant 3). Together, these peptides were found in 262 (75.1%) of the isolates. The fHbp peptide diversity, their occurring frequencies and association with major CC are shown in Table 3. Although the majority (88.1%) of MenB CC269 were predicted to produce fHbp variant 1 peptide 15, 56.0% of the CC41/44 were predicted to produce either fHbp variant 1 peptide 4 (24%) or variant 2 peptide 19 (32%). Twelve (85.7%) of fourteen CC213 were predicted to produce fHbp variant 3 peptide 45.

**TABLE 3:** fHbp peptide types: frequency of occurrence and association with clonal complexes in 349 invasive serogroup B N. meningitidis in Canada, 2010 to 2014

Peptide type^*a*^	No. (%) of isolates^*b*^	No. of major CCs associated
Total variant 1	236 (67.6)	
15	148 (42.4)	All CC269
4	35 (10.0)	34 CC41/44
13	16 (4.6)	5 CC1157; 4 CC60
1	7 (2.0)	6 unassigned^*c*^
14	6 (1.7)	5 CC41/44
Others^*d*^	24 (6.9)	Various
		
Total variant 2	87 (24.9)	
19	40 (11.5)	32 CC41/44; 7 CC269
16	11 (3.2)	All CC35
24	9 (2.6)	6 CC41/44
106	7 (2.0)	6 unassigned
23	4 (1.1%)	3 CC41/44
Others^*e*^	16 (4.6)	Various
		
Total variant 3	24 (6.9)	
45	12 (3.4)	All CC213
Others^*f*^	12 (3.4)	Various

a A total of 50 peptide types were identified (23 variant 1, 17 variant 2, and 10 variant 3).

b Two isolates did not encode factor H binding protein (fHbp): one with *fhbp* gene allele 743 (internal stop codon) and one with gene allele 755 (frameshift mutation).

c Unassigned to any known clonal complex in the MLST website.

d Other variant 1 fHbp peptides = three each of peptide 410 and 509; two each of peptides 54 and 210; and one each of peptides 2, 12, 100, 108, 224, 233, 249, 256, 322, 626, 628, 631, 677, and 687.

e Other variant 2 fHbp peptides = three of peptide 506; two each of peptides 21 and 109; and one each of peptides 22, 25, 49, 102; 118, 527, 625, 632, and 656.

f Other variant 3 fHbp peptides = two each of peptides 31, 47, and 160 and one each of peptides 30, 98, 177, 555, 627, and 688.

Of the 43 NHBA peptide types identified, the five most common peptides (2, 6, 18, 21, and 112) were found in 253 isolates (72.5%), and 21 peptide types occurred only once (found in single isolate). The NHBA diversity, their occurring frequencies and association with major CC are described in Table 4. While 68.0% of the MenB CC 41/44 were found to have *nhba* genes to encode either NHBA peptide 2 (41.0%) or NHBA peptide 112 (27.0%), 88.2% of the CC269s were encoding NHBA peptide 21.

**TABLE 4:** *Neisseria* heparin binding antigen peptide types: frequencies of occurrences and association with major clonal complexes in 349 invasive serogroup B N. meningitidis in Canada, 2010 to 2014

NHBA peptides	No. (%) of isolates	Major CC(s) associated^*a*^
2	41 (11.7)	All CC41/44
6	12 (3.4)	5 CC269; 1 CC41/44; 6 unassigned
18	11 (3.2)	All CC213
112	27 (7.7)	All CC41/44
21	162 (46.4)	148 CC269; 11 CC35; 3 unassigned
Others^*b*^	96 (27.5)	Various

a Unassigned, unassigned to any known clonal complex in the MLST website.

b Other *Neisseria* heparin binding antigen (NHBA) peptide types = eight each of peptides 3, 10, and 29; six each of peptides 24 and 47; five each of peptides 5, 20, 114, and 122; four of peptide 770; three of peptide 276; two each of peptides 7, 43, 101, 279, 768, and 775; and 21 peptides that occurred only once each (single isolates).

Of the 349 MenB isolates tested, 316 (90.5%) did not give a PCR amplicon with PCR primers that targeted the *nadA* gene. Of the 33 isolates with *nadA* genes detected, 19 had mutations that prevented NadA protein synthesis, 13 were predicted to encode variant 1 peptides (10 for peptide 1, 2 for peptide 100, and 1 for peptide 143), and 1 was predicted to encode a variant 2/3 peptide (peptide 3). Twelve (92.3%) of the thirteen isolates predicted to produce NadA variant 1 peptides belonged to the CC32, and the remaining isolate belonged to the CC41/44. A MenB of ST336 (unassigned to any known CC) was predicted to produce a variant 2/3 NadA peptide 3.

The most common PorA VR1 variants were 7-2 (57 isolates), 18-7 (44 isolates), 19-11 (126 isolates), and 22 (32 isolates). The most common PorA VR2 variants were 4 (40 isolates), 9 (48 isolates), 14 (31 isolates), and 15-11 (126 isolates). The occurring frequencies of different PorA VR1/VR2 genotypes and their associations with major CCs are described in Table S2. The PorA genotype P1.19-1,15-11 was found in the majority (74.4%) of the MenB CC269. Of the 14 MenB CC213, 13 (93.0%) were found to have PorA genotype P1.22,14, and 37% of the CC41/44 were found to have PorA genotype P1.7-2,4.

10.1128/mSphere.00883-19.2 TABLE S2 Outer membrane protein PorA VR1, VR2 variants, and PorA genotypes (VR1/VR2): frequency of occurrence and association with clonal complexes (CCs) in 349 invasive serogroup B N. meningitidis in Canada, 2010 to 2014. Download Table S2, PDF file, 0.2 MB. © Crown copyright 2020. 2020 Crown This content is distributed under the terms of the Creative Commons Attribution 4.0 International license.

### Estimated coverage of Canadian MenB isolates by 4CMenB vaccine as determined by meningococcal antigen typing system (MATS).

The overall predicted coverage of the 250 MenB isolates across Canada was 184/250 (73.6%) [95% CI, 54% to 85%]. Predicted coverage according to the major MenB CC is described in Table 5. The estimated coverages by the number of antigens were as follows: 32.0% of isolates were covered by one antigen, 29.2% by two antigens, and 12.4% by three antigens, but no strain was covered by all four antigens (Table S3). MenB strains with PorA antigen P1.4 (40 isolates) were also found to have one or more other 4CMenB antigens detected by the meningococcal antigen typing system (MATS) at relative potency (RP) levels above the positive bactericidal threshold (PBT) and hence predicted to be covered by these non-PorA antigens. More than 40% (41.6%) of the covered strains were covered by two or more antigens. (Table S3). The estimated coverage conferred by each antigen was as follows: fHbp = 62.0% (95% confidence interval [CI], 46 to 65%), NHBA = 48.4% (95% CI, 19 to 47%), PorA = 15.6% (40 isolates were typed as PorA P1.4), and NadA = 1.2% (95% CI, 0.4 to 2.0%). The predicted coverage varied between provinces: it ranged from 85.7% in the Atlantic region and 84.3% in Quebec to 59.0% in Ontario and 60.9% in British Columbia (Table 5).

**TABLE 5:** Meningococcal antigen typing system determined potential coverage of 4CMenB vaccine on a panel of 250 invasive serogroup B N. meningitidis (MenB) isolates in Canadian provinces from 2010 to 2014 according to clonal complexes

Parameter	No. (%) of isolates predicted to be covered/no. tested^*a*^						
	BC	AB	Central	ON	QC	Atlantic	All provinces
No. of isolates tested	23	18	18	61	102	28	250
No. predicted to be covered	14 (60.9)	13 (72.2)	11 (61.1)	36 (59.0)	86 (84.3)	24 (85.7)	184 (73.6)
MenB CC							
41/44	9/10 (90.0)	5/7 (71.4)	4/4 (100.0)	16/23 (69.6)	8/20 (40.0)	20/21 (95.2)	62/85 (72.9)
269	3/4 (75.0)	2/2 (100.0)	3/3 (100.0)	5/11 (45.5)	70/73 (95.9)	2/3 (66.7)	85/96 (88.5)
32	1/1 (100.0)	3/3 (100.0)	0	4/6 (66.7)	2/2 (100.0)	1/1 (100.0)	11/13 (84.6)
35	0	1/1 (100.0)	2/2 (100.0)	2/6 (33.3)	2/2 (100.0)	0	7/11 (63.6)
37	0	0	0	0	1/1 (100.0)	0	1/1 (100.0)
60	0	0	0	2/2 (100.0)	0	1/1 (100.0)	3/3 (100.0)
162	0			1/1 (100.0)	1/1 (100.0)	0	3/3 (100.0)
213	0/6	0/2	0/3	0/2	0	0	0/13
461	0	0	0/1	0/1	0	0	0/13
865	0	0	1/1	1/1 (100.0)	0	0	2/2 (100.0)
1157	0	1/1 (100.0)	0	1/2 (50.0)	0	0	2/3 (66.7)
							
None assigned^*b*^	1/2 (50.0)	0/1	1/4 (25.0)	4/6 (66.7)	2/3 (66.7)	0/2	8/14 (44.4)

a The Central region includes Saskatchewan and Manitoba; the Atlantic region includes New Brunswick, Nova Scotia, Prince Edward Island, Newfoundland, and Labrador. BC, British Colombia; AB, Alberta; ON, Ontario; QC, Quebec.

b None assigned, sequence types not grouped into any known CC.

10.1128/mSphere.00883-19.3 TABLE S3 Number of serogroup B N. meningitidis (MenB) isolates predicted to be covered by 4CMenB vaccine antigen combination according to clonal complexes (CC). Download Table S3, PDF file, 0.01 MB. © Crown copyright 2020. 2020 Crown This content is distributed under the terms of the Creative Commons Attribution 4.0 International license.

The predicted coverage of CC269 isolates was 88.5% (85 of 96 isolates) and that for CC41/44 was 72.9% (62 of 85 isolates) (Table 5). Predicted coverage of the CC41/44 MenB isolates by the 4CMenB vaccine was mainly through the PorA, fHbp, and NHBA components, while coverage of the CC269 isolates was mainly through fHbp and NHBA. Predicted coverage of the CC32 isolates was 84.6% and mainly through the fHbp and NHBA components. The predicted coverage of the CC35 isolates was 63.6% with most protection afforded by the NHBA component. In contrast, predicted coverage of the fourteen CC213 isolates was zero. Of the most common MenB STs, their coverage as estimated by MATS is shown in Table S4. It ranged from a high of 100% for ST154 and 95.9% for ST269 to a low of 0% for ST213 and 16.6% for ST5571.

10.1128/mSphere.00883-19.4 TABLE S4 Meningococcal antigen typing system (MATS) determined the potential coverage of 4CMenB vaccine on a panel of 250 invasive serogroup B N. meningitidis (MenB) isolates in Canada, 2010 to 2014, according to the most frequently identified sequence type (ST) and the predicted protective antigen combination. Download Table S4, PDF file, 0.01 MB. © Crown copyright 2020. 2020 Crown This content is distributed under the terms of the Creative Commons Attribution 4.0 International license.

**FIG 1:**
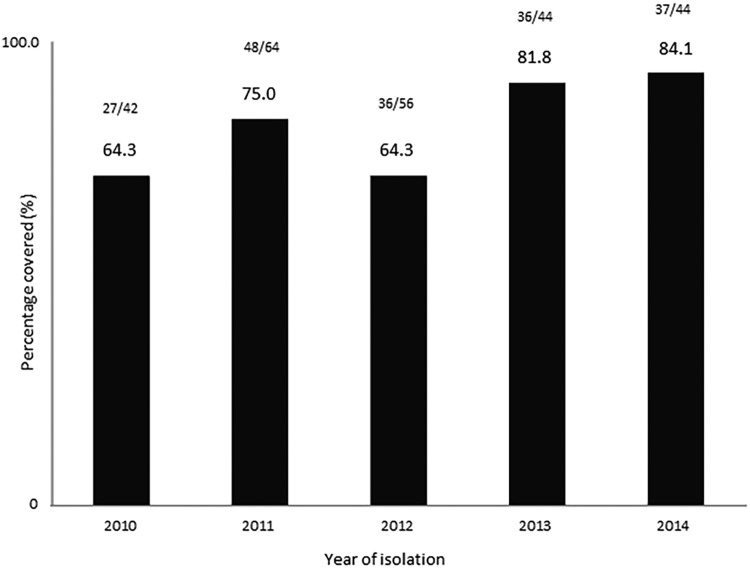
Estimated 4CMenB coverage of 250 invasive N. meningitidis serogroup B isolates by calendar year in Canada, 2010 to 2014, determined by the MATS assay.

**FIG 2:**
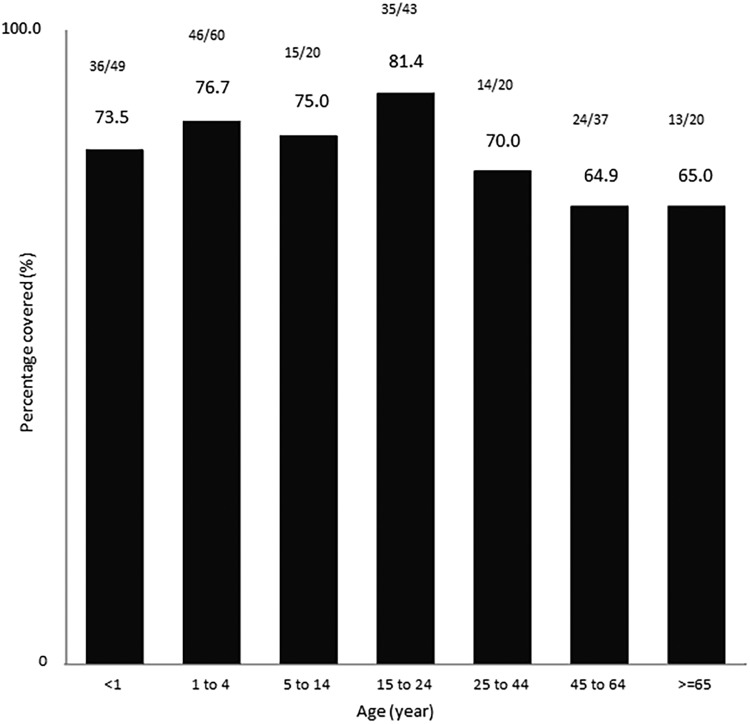
Estimated 4CMenB coverage of 250 invasive N. meningitidis serogroup B isolates recovered from patients of different age groups and tested by the MATS assay. (One patient isolate had no age information.)

Predicted coverages estimated by year for the 5-year period from 2010 to 2014 were 64.3, 75.0, 64.3, 81.8, and 84.1%, respectively (Fig. 1). The predicted coverage by age group is shown in Fig. 2. The highest coverage was for strains recovered from patients aged 15 to 24 years (81.4%) while the lowest coverage was for strains recovered from patients aged 45 to 64 years and those older than 65 years (64.9 to 65.0%, respectively). Overall, the estimated coverage was 75.2% for infants and children ≤4 years old and 72.1% for those ≥5 years old (Fig. 2).

## DISCUSSION

In this study, we examined culture-confirmed IMD case isolates obtained from across Canada between 2010 and 2014. First, we showed a drop of 38.4% in the number of cases since 2011. Second, the number of cases from the different provinces also varied, which translated into geographical variations in the prevalence of culture confirmed IMD. The highest mean annual rate of culture-confirmed IMD was found in Quebec and New Brunswick (0.59/100,000 and 0.50/100,000, respectively), while the lowest rate was in Ontario (0.19/100,000), and the mean annual rate for Canada overall was 0.31/100,000. This estimation of the order of prevalence of disease agreed very well with published data. The overall prevalence (annual rate per 100,000 population) of IMD in Canada during this study period (2010 to 2014) was between 0.45 and 0.28, with an average rate of 0.40/100,000 (15). The average rate of IMD reported in Quebec from 2006 to 2013 was 0.7/100,000, which was 1.8 times higher than the national average, but in the region of Saguenany-Lac-Saint-Jean, the rate was 5 times higher than the provincial average and 8.5 times higher than the national average (16). In contrast, the annual rates in Ontario during 2010 to 2014 ranged from 0.3 to 0.2, with an average rate of 0.26/100,000 (17), which was 1.5 times lower than the national average. The published rates from New Brunswick were 0.7, 0.5, 0.8, 0.3, 0.3, and 0.7/100,000 from 2010 to 2015, respectively, with a mean annual rate of 0.54/100,000 (18). Therefore, data based on IMD isolates received at the NML did suggest that our laboratory surveillance program provided a good overall picture of IMD in Canada. As such, the IMD isolates in our collection represented a true and unbiased sample of invasive N. meningitidis strains causing IMD in Canada.

This study also showed a significant geographical variation in the serogroup distribution of IMD case isolates. In the Atlantic region and Quebec, MenB was significantly more common than other serogroups combined, while in the rest of the country, there was no such significant difference. Another interesting observation was the differential age distributions of cases due to MenB, MenC, and MenY. The majority of the MenC and MenY cases (75.0 and 69.6%, respectively) were in adults over the age of 24 years. In contrast, 67.2% of MenB cases were in those aged 24 years and under. This may be explained by the implementation of the MenC conjugate vaccine programs in infants and/or the MenACWY conjugate vaccine program in high school children, as well as in some provinces in the infant program (9).

Since Canada currently has two licensed MenB vaccines but no universal MenB vaccination program despite its prevalence as a cause of IMD, we characterized the MenB isolates collected from our national laboratory IMD surveillance program for the period 2010 to 2014. Overall, the MenB population in Canada was genetically very diverse, with 103 different STs identified, 90 of which were grouped into 13 CCs. However, over three-quarters (268 isolates or 76.8%) of 349 MenB were grouped as either CC269 (168 isolates or 48.1%) or CC41/44 (100 isolates or 28.7%). Also, isolates of CC269 with 14 different STs identified were relatively more clonal (diversity index, 0.285), whereas isolates of CC41/44 with 46 different STs identified were significantly more diverse (diversity index, 0.909). This difference in the genetic diversity of these two major CCs was confirmed by the relatively larger number of 20 fHbp and 17 NHBA peptide types encoded by isolates of the CC41/44 compared to the relatively smaller number of 11 fHbp and 11 NHBA peptide types encoded by the CC269 isolates.

In Quebec, the hyperendemic IMD condition was driven by the emergence and persistence of the hypervirulent MenB clone of ST269 (19). In the neighboring province of Ontario, IMD occurred at a low endemic level typically caused by a diverse variety of strains as shown previously (20) and in this study. Indeed, the genetic diversity index of the MenB population in Ontario was determined to be much higher (0.9858) than the indexes found for MenB strains in Quebec (0.5588) and the Atlantic region (0.3651), confirming the hyperendemic MenB disease conditions in these two regions of Canada. In New Brunswick the MenB diversity index was 0.323, which matched the higher incidence rate of culture confirmed IMD in this province (0.50/100,000), compared to the whole Atlantic region (0.26/100,000). The diversity indexes (0.9542 to 0.9684) found for MenB in Western Canada also suggested endemic MenB disease and a low level of IMD.

Although MenB was the most common serogroup described in this study and also in many European countries (21), their clonal nature also showed geographical variations. In Canada, the most common MenB CCs in isolates obtained from 2010 to 2014 were CC269 (48.1%), CC41/44 (28.7%), CC213 (4.0%), and CC32 (4.0%). In England, Wales, and Northern Ireland, their corresponding frequencies from 2007 to 2008 and 2014 to 2015 were 32 to 24% for CC269, 32 to 33% for CC41/44, 10% in both periods for CC213, and 6 to 9% for CC32 (22). In Spain, from a panel of 300 representative MenB isolates obtained from 2009 to 2010 (23), the five most common CCs and their frequencies were CC269 (18%), CC213 (17%), CC32 (16%), CC461 (8%), and CC41/44 (7%). In Portugal, the frequencies of isolates belonging to CC269, CC41/44, CC213, and CC162 were 13.2, 31.1, 14.2, and 7.5%, respectively (24). Not only were the frequencies of the different CCs varied between countries, the actual strain type as identified by their STs were also different between countries. In Canada, the predominant strain in the CC269 was ST269 (84.5%), with the majority typed with PorA genotype of P1.19-1,15-11, which is predicted to have fHbp peptide 1.15 and NHBA peptide 21 and lack NadA. In England, Wales, and Northern Ireland, the CC269 was divided into two clusters that were represented by ST269 and ST275, each with their own unique PorA, fHbp, and NHBA types (22). In Spain (23), the predominant strain type of CC269 was ST1163 (63% of CC269), a member of the ST275 cluster (25). Neither ST275 nor ST1163 was identified in our present study of Canadian MenB. Two isolates of ST275 had been identified in Quebec in an early study that examined 180 MenB isolates of CC269 collected from 2003 to 2010 (19).

Considering the geographical variations in disease prevalence, serogroup distribution, and MenB strain characteristics, we selected a representative sample of MenB isolates (71.6% of the whole MenB collection from this study) for the strain coverage estimation offered by the licensed 4CMenB vaccine. The current MATS data showed that the unique regional IMD epidemiology appeared to affect the predicted 4CMenB coverage. For example, the highest coverages (85.7 and 84.3%, respectively) were obtained for strains recovered from the Atlantic region and Quebec, and the lowest coverage (59.0%) was observed for strains recovered from Ontario; coverages for British Columbia, Alberta, and Saskatchewan/Manitoba were between 60.9 and 72.2%. Regional differences in 4CMenB strain coverage had also been reported in the United Kingdom, with differences ranging from 53 to 79% for strains obtained during 2007 and 2008 and from 48 to 80% for strains recovered during 2014 and 2015 (22). The highest strain coverage by 4CMenB observed in the Atlantic region and Quebec was probably related to the predominance of ST154 and ST269, respectively, in these two regions. The 4CMenB coverage estimated in this study for ST269 was 95.9%, while for ST154 it was 100%, which was very similar to previous estimates of 95 and 100%, respectively, for these two STs done on strains obtained from 2006 to 2009 (14). The consistency of the MATS coverage on these two STs may suggest a stability of these two genotypes of meningococci over the 9-year period (2006 to 2014) to express the 4CMenB vaccine antigens.

Besides provincial or regional differences observed in vaccine strain coverage, differences in vaccine coverage on distinct MenB clones within a province or region were also evident in this study. For example, in Quebec, the strain coverage for ST269 was 96.9% (63 of 65 isolates tested), but for ST571 the coverage was 42.9% (three of seven isolates tested). Therefore, despite the predominance of ST269 in Quebec, the overall MenB strain coverage in Quebec was only 84.3%, probably because some strain type, such as ST571, was poorly covered by the vaccine. In British Columbia, due to the small number of MenB isolates detected, coverage by ST was not feasible, and instead coverage by CC was compared. The strain coverage there for the CC41/44 was 90.0% (nine of ten isolates tested), while coverage for CC213 was 0% (0 of six isolates tested). The CC213 represented 26.1% of the MenB in British Columbia, and its failure to be covered by the 4CMenB vaccine was reflected in the overall MenB strain coverage of 60.9% in this province.

Our MATS data showed that 4CMenB coverage on Canadian MenB strains appeared to be higher for strains collected in 2013 and 2014 (mean coverage of 83.0%) compared to strains collected from 2010 to 2012 (mean coverage of 67.7%). Also, coverage for MenB strains recovered from infants, children, and young adults (≤24 years old), who represented two-thirds of all MenB IMD cases in Canada, was higher (73.0 to 81.0%) compared to strains collected from those aged 45 years and above (65.0%). Also, the predicted coverage for Canadian MenB strains appeared to be comparable with data obtained globally (26). The higher predicted coverage of MenB strains in the United States (91.0%) and in Canada in 2013 to 2014 (83.0%) compared to data from the United Kingdom (66%), Portugal (68%), and Spain (69%) may be related to the unique epidemiology of MenB IMD in North America. In contrast to these European countries, which have not been affected by any predominant MenB clone, both the United States and Canada have their MenB epidemiology dominated by strains of either CC32 (in the United States) or CC269 (in Canada). The predicted 4CMenB coverages of these two CCs were very high. In the United States, the predicted coverage of MenB strains belonging to CC32 was 99% (27); in Canada, the coverage of MenB belonging to CC269 was 88.5%, and for the predominant strain of ST269 it was 95.9% (this study). Indeed, in a field use of the 4CMenB vaccine in one region of Quebec, it was shown that the direct vaccine protection rate was 79% and that the overall impact of the vaccine was 86% in the reduction of MenB IMD risk (28). MATS has been developed as a conservative tool, which underestimates coverage, particularly in older age groups. The choice to apply PBTs derived on infant sera to all age groups was intentionally made to avoid overestimation of coverage. A consequence of that choice was some level of underestimation, as demonstrated by the observation that strains that were killed in serum bactericidal assay using human complement (hSBA) were not always predicted to be killed by MATS, particularly in older ages. However, results reported by Frosi et al. (29) and by Stella et al. (30) showed that MATS provide accurate and conservative estimates of strain coverage for a strain panel representative of IMD in a specific geographic setting and epidemiologic year. Therefore, MATS remains a useful tool for ongoing postlicensure surveillance of the effectiveness of 4CMenB and to monitor epidemiological trends on the expression of the vaccine antigens.

In conclusion, we have shown an overall decrease of IMD in Canada over the period 2010 to 2014, as a result of MenC and MenACWY conjugate vaccine programs and a natural cycle of fewer MenB disease. However, there were regions (some area in Quebec and New Brunswick) with higher rates of IMD due to certain MenB strain types than the rest of the country. Geographical variations in serogroup distribution and unique MenB strain types were probably affecting the predicted coverage of the 4CMenB vaccine in different regions of Canada. Therefore, our data support the current Canadian guidelines on the use of 4CMenB vaccine to control outbreaks and increased incidence of MenB disease when the responsible strain has been predicted to be covered by the vaccine (31). The temporal and geographical variations in MenB strain types and prevalence of MenB disease within a country and globally makes laboratory surveillance, particularly the characterization of strains, including their susceptibility to MenB vaccine-induced antibodies, an important public health priority.

## MATERIALS AND METHODS

Invasive N. meningitidis isolates (defined by isolation from a normally sterile body site) are routinely sent by the provincial and territorial public health laboratories in Canada to the National Microbiology Laboratory (NML) as part of the national surveillance program (8). This study made use of invasive N. meningitidis isolates received by the NML between the period of 1 January 2010 to 31 December 2014. All isolates included in this study were from individual IMD cases, and when more than one isolate was received from the same patient within a 2-week period, only one isolate was included in the study. Demographic data on the case isolates (province of isolation and age of cases) were obtained from the specimen requisition forms.

The identity of each isolate was confirmed by biochemical tests, and its serogroup was determined by slide agglutination using rabbit anti-grouping antisera and confirmed by PCR if the strain was nonagglutinable or autoagglutinable (19). Clonal analysis was determined by MLST (32). DNA sequencing of their *porA*, *fHbp*, *nhba*, and *nadA* genes and nomenclature of their PorA genotype, as well as their fHbp, NHBA, and NadA peptide types, was done as previously described (33). Primers used for PCR were described in Table S5. To determine the genetic diversity of the MenB populations in the different provinces and in the major CCs, the Simpson’s diversity index (0 to 1) was determined (34), with values nearer 1 reflecting more diversity and values nearer 0 reflecting less diversity.

10.1128/mSphere.00883-19.5 TABLE S5 PCR and sequencing primers used in this study. Download Table S5, PDF file, 0.04 MB. © Crown copyright 2020. 2020 Crown This content is distributed under the terms of the Creative Commons Attribution 4.0 International license.

The expression levels and degree of cross-reactivities of the 4CMenB vaccine components FHbp, NHBA, and NadA in N. meningitidis clinical isolates were determined by a meningococcal antigen typing system (MATS) assay to provide an estimation of the predicted coverage conferred by the 4CMenB vaccine on the MenB test strains. Bacterial strains were cultured on chocolate agar plates and incubated over night at 37°C, 5% CO_2_. Single colonies were collected from chocolate agar plates and suspended in Mueller-Hinton broth to get an optical density of 0.4. A detergent (Empigen BB 5%) was added to the suspensions to release the antigens and inactivate bacteria. Bacterial lysates were added onto three microwell plates, coated with polyclonal antibodies derived from the three antigens (for fHbp; NHBA and NadA), and incubated for 1 h. After washing, biotin-labeled antibodies were added to the plates, followed by incubation for 1 h. Horseradish peroxidase-conjugated streptavidin was added to the plates after washing, followed by incubation for 30 min. An OPD substrate was added and developed for 20 min before blocking the reaction with H_2_SO_4_, and plates were read on a microplate reader. The relative difference estimated by comparing the titration curves of the unknown sample to the titration curve of the reference strain was defined as the relative potency (RP) (35, 36). Coverage was defined by comparing the RP values generated for each antigen by MATS on the test strain to the minimum level of RP known as the positive bactericidal threshold (PBT) established for each corresponding antigen (35). The PBT values were set at 29.4% for NHBA, 0.9% for NadA, and 1.2% for FHbp (35, 37). A strain was considered covered for an antigen if a higher RP value (defined as positive) was obtained compared to the PBT threshold value set for that particular antigen. In addition, strains determined to contain the PorA P1.4 antigen, by DNA sequencing their *porA* gene, would be considered covered by the OMV P1.4 component of the vaccine. Therefore, by combining MATS and *porA* sequencing results, strains were grouped into four categories: covered by one antigen only (either only one positive RP value was obtained from MATS or *porA* P1.4 present), covered by two antigens (either any two positive RP values were obtained or one positive RP value plus *porA* P1.4 is present), covered by three antigens (either three positive RP values or two positive RP value plus *porA* P1.4 are present), or covered by all four antigens (if three positive RP values plus *porA* P1.4 are present).

Since the PBT was strain independent, an empirical estimate of 95% CI around each PBT was derived with a log normal approximation based on overall assay reproducibility, assessed during MATS interlaboratory standardization study (38). The 95% CIs of the PBT were used to define the 95% CIs of strain coverage (0.169 to 0.511 for NHBA, 0.004 to 0.019 for NadA, and 0.008 to 0.018 for FHbp).

From the 349 MenB isolates received from across Canada during 2010 to 2014, 250 were selected for the MATS assay, and these included 102 (50.7%) MenB from Quebec and 148 isolates that represented all the MenB received from the rest of Canada (Table S1). The decision not to test all the MenB from Quebec was based on the fact that many (72.1%) of the MenB were determined to be clonal (19).

A Student *t* test was done using the Excel program in Microsoft Office 2016 (https://www.rwu.edu/sites/default/files/downloads/fcas/mns/running_a_t-test_in_excel.pdf).

Population estimates by province in Canada for 2014 were obtained from Statistics Canada (39).
