# Supplementary material for: Culture-Confirmed Invasive Meningococcal Disease in Canada, 2010 to 2014: Characterization of Serogroup B Neisseria meningitidis Strains and Their Predicted Coverage by the 4CMenB Vaccine
Source: mSphere. 2020 Mar 4;5(2):e00883-19. doi: 10.1128/mSphere.00883-19 (PMC7056808; doi:10.1128/mSphere.00883-19)
Supplement: TABLE S1 [file mSphere.00883-19-st001.pdf]

**Supplementary Table S1.**

| | Number of isolates (provincial total in brackets) according to provinces and STs grouped by CCs | | | | | | |
|---|---|---|---|---|---|---|---|
| CC :<br>ST | BC (23) | AB (18) | SK (6) MB (12)<br>Central (18) | ON (61) | QC (201) | PE (1) NS (5) NL (5) NB (17)<br>Atlantic (28) | Canada |
| 41/44 : | 10 | 7 | 4 | 23 | 35 | 21 | 100 |
| 41 | 0 | 0 | 0 | 1 | 2 | 0 | 3 |
| 42 | 0 | 0 | 0 | 1 | 0 | 0 | 1 |
| 44 | 0 | 1 | 0 | 1 | 0 | 0 | 2 |
| 110 | 0 | 1 | 0 | 0 | 0 | 0 | 1 |
| 136 | 2 | 0 | 0 | 0 | 1 | 0 | 3 |
| 154 | 1 | 1 | 3 | 3 | 1 | 17 | 26 |
| 207 | 0 | 0 | 0 | 1 | 0 | 1 | 2 |
| 315 | 0 | 0 | 0 | 0 | 1 | 0 | 1 |
| 340 | 1 | 0 | 0 | 0 | 0 | 0 | 1 |
| 409 | 1 | 0 | 0 | 0 | 0 | 1 | 2 |
| 571 | 0 | 0 | 0 | 2 | 13 | 0 | 15 |
| 944 | 0 | 0 | 0 | 1 | 0 | 0 | 1 |
| 1166 | 0 | 0 | 0 | 1 | 0 | 0 | 1 |
| 1194 | 0 | 0 | 0 | 1 | 0 | 0 | 1 |
| 1473 | 1 | 0 | 0 | 0 | 0 | 0 | 1 |
| 1833 | 0 | 0 | 0 | 0 | 1 | 0 | 1 |
| 2288 | 1 | 0 | 0 | 0 | 0 | 0 | 1 |
| 2314 | 0 | 0 | 0 | 1 | 0 | 0 | 1 |
| 2820 | 0 | 0 | 0 | 0 | 4 | 0 | 4 |
| 5110 | 0 | 0 | 0 | 1 | 0 | 0 | 1 |
| 5550 | 0 | 1 | 0 | 0 | 0 | 0 | 1 |
| 5553 | 0 | 1 | 0 | 0 | 0 | 0 | 1 |
| 5554 # | 0 | 0 | 0 | 0 | 1 | 0 | 1 |
| 6058 | 1 | 0 | 0 | 0 | 0 | 0 | 1 |
| 6169 | 0 | 0 | 0 | 2 | 0 | 0 | 2 |
| 6617 | 1 | 0 | 0 | 3 | 0 | 0 | 4 |
| 7612 | 0 | 0 | 0 | 0 | 0 | 1 | 1 |
| 8052 # | 0 | 0 | 0 | 0 | 1 | 0 | 1 |
| 8769 # | 0 | 0 | 0 | 0 | 1 | 0 | 1 |
| 8771 | 0 | 0 | 0 | 1 | 0 | 0 | 1 |
| 8925 | 0 | 0 | 0 | 0 | 1 | 0 | 1 |
| 9171 | 0 | 0 | 1 | 0 | 0 | 0 | 1 |

| | | | | | | | | |
|---|---|---|---|---|---|---|---|---|
| 9352 | 0 | 1 | 0 | 0 | 0 | 0 | | 1 |
| 9411 | 0 | 0 | 0 | 0 | 0 | 1 | | 1 |
| 9415 | 0 | 0 | 0 | 2 | 0 | 0 | | 2 |
| 9416 # | 0 | 0 | 0 | 0 | 1 | 0 | | 1 |
| 10185 | 0 | 0 | 0 | 0 | 1 | 0 | | 1 |
| 10187 | 0 | 0 | 0 | 1 | 0 | 0 | | 1 |
| 10324 # | 0 | 0 | 0 | 0 | 1 | 0 | | 1 |
| 10329 | 0 | 0 | 0 | 0 | 1 | 0 | | 1 |
| 10330 | 1 | 0 | 0 | 0 | 0 | 0 | | 1 |
| 10347 | 0 | 1 | 0 | 0 | 0 | 0 | | 1 |
| 10588 # | 0 | 0 | 0 | 0 | 1 | 0 | | 1 |
| 10619 | 0 | 0 | 0 | 0 | 1 | 0 | | 1 |
| 10658 | 0 | 0 | 0 | 0 | 1 | 0 | | 1 |
| 11009 | 0 | 0 | 0 | 0 | 1 | 0 | | 1 |
| 269: | 4 | 2 | 3 | 11 | 145 | 3 | | 168 |
| 13 | 0 | 0 | 0 | 2 | 1 | 0 | | 3 |
| 269 | 1 | 2 | 2 | 4 | 133 | 0 | | 142 |
| 479 | 1 | 0 | 1 | 0 | 0 | 0 | | 2 |
| 565 | 0 | 0 | 0 | 1 | 1 | 0 | | 2 |
| 1161 | 0 | 0 | 0 | 0 | 0 | 1 | | 1 |
| 1214 | 0 | 0 | 0 | 1 | 0 | 0 | | 1 |
| 1986 | 0 | 0 | 0 | 0 | 2 | 0 | | 2 |
| 2738 | 0 | 0 | 0 | 1 | 1 | 0 | | 2 |
| 2976 | 1 | 0 | 0 | 0 | 0 | 0 | | 1 |
| 5494 | 1 | 0 | 0 | 2 | 3 | 0 | | 6 |
| 8924 | 0 | 0 | 0 | 0 | 1 | 2 | | 3 |
| 8772# | 0 | 0 | 0 | 0 | 1 | 0 | | 1 |
| 8880# | 0 | 0 | 0 | 0 | 1 | 0 | | 1 |
| 11008 | 0 | 0 | 0 | 0 | 1 | 0 | | 1 |
| 213: | 6 | 2 | 3 | 2 | 1 | 0 | | 14 |
| 213 | 4 | 0 | 3 | 2 | 0 | 0 | | 9 |
| 3123 | 2 | 0 | 0 | 0 | 0 | 0 | | 2 |
| 9413 | 0 | 2 | 0 | 0 | 0 | 0 | | 2 |
| 3113# | 0 | 0 | 0 | 0 | 1 | 0 | | 1 |
| 32: | 1 | 3 | 0 | 6 | 3 | 1 | | 14 |
| 32 | 1 | 3 | 0 | 2 | 2 | 0 | | 8 |
| 290 | 0 | 0 | 0 | 0 | 0 | 1 | | 1 |
| 2726 | 0 | 0 | 0 | 0 | 1 | 0 | | 1 |
| 6544 | 0 | 0 | 0 | 2 | 0 | 0 | | 2 |
| 7301 | 0 | 0 | 0 | 1 | 0 | 0 | | 1 |
| 9410 | 0 | 0 | 0 | 1 | 0 | 0 | | 1 |
| 35: | 0 | 1 | 2 | 6 | 4 | 0 | | 13 |

| | | | | | | | |
|---|---|---|---|---|---|---|---|
| 35 | 0 | 1 | 0 | 1 | 0 | 0 | 2 |
| 278 | 0 | 0 | 0 | 0 | 2 | 0 | 2 |
| 564 # | 0 | 0 | 0 | 0 | 1 | 0 | 1 |
| 790 | 0 | 0 | 1 | 0 | 0 | 0 | 1 |
| 3626 # | 0 | 0 | 0 | 0 | 1 | 0 | 1 |
| 6615 | 0 | 0 | 0 | 3 | 0 | 0 | 3 |
| 10183 | 0 | 0 | 0 | 1 | 0 | 0 | 1 |
| 10186 | 0 | 0 | 0 | 1 | 0 | 0 | 1 |
| 10657 | 0 | 0 | 1 | 0 | 0 | 0 | 1 |
| 37: | 0 | 0 | 0 | 0 | 1 | 0 | 1 |
| 917 | 0 | 0 | 0 | 0 | 1 | 0 | 1 |
| 60: | 0 | 0 | 0 | 2 | 1 | 1 | 4 |
| 60 | 0 | 0 | 0 | 2 | 0 | 0 | 2 |
| 11011 | 0 | 0 | 0 | 0 | 0 | 1 | 1 |
| 10587 # | 0 | 0 | 0 | 0 | 1 | 0 | 1 |
| 103: | 0 | 0 | 0 | 0 | 1 | 0 | 1 |
| 4963 # | 0 | 0 | 0 | 0 | 1 | 0 | 1 |
| 162: | 0 | 1 | 0 | 1 | 1 | 0 | 3 |
| 162 | 0 | 1 | 0 | 0 | 1 | 0 | 2 |
| 10323 | 0 | 0 | 0 | 1 | 0 | 0 | 1 |
| 254: | 0 | 0 | 0 | 0 | 1 | 0 | 1 |
| 10332 # | 0 | 0 | 0 | 0 | 1 | 0 | 1 |
| 461: | 0 | 0 | 1 | 1 | 1 | 0 | 3 |
| 461 | 0 | 0 | 1 | 1 | 1 | 0 | 3 |
| 865: | 0 | 0 | 1 | 1 | 0 | 0 | 2 |
| 3327 | 0 | 0 | 1 | 1 | 0 | 0 | 2 |
| 1157: | 0 | 1 | 0 | 2 | 2 | 0 | 5 |
| 1157 | 0 | 1 | 0 | 2 | 2 | 0 | 5 |
| Unassigned: | 2 | 1 | 4 | 6 | 5 | 2 | 20 |
| 336 | 0 | 0 | 1 | 1 | 1 | 0 | 3 |
| 897 | 0 | 0 | 0 | 1 | 0 | 0 | 1 |
| 938 # | 0 | 0 | 0 | 0 | 1 | 0 | 1 |
| 3986 # | 0 | 0 | 0 | 0 | 1 | 0 | 1 |
| 5571 | 1 | 0 | 0 | 2 | 1 | 2 | 6 |
| 7744 | 0 | 0 | 1 | 0 | 0 | 0 | 1 |
| 8894 | 0 | 1 | 0 | 0 | 0 | 0 | 1 |

| | | | | | | | |
|---|---|---|---|---|---|---|---|
| 9248 | 0 | 0 | 0 | 0 | 1 | 0 | 1 |
| 9409 | 0 | 0 | 0 | 1 | 0 | 0 | 1 |
| 9412 | 0 | 0 | 1 | 0 | 0 | 0 | 1 |
| 10184 | 1 | 0 | 0 | 0 | 0 | 0 | 1 |
| 10423 | 0 | 0 | 0 | 1 | 0 | 0 | 1 |
| 11018 | 0 | 0 | 1 | 0 | 0 | 0 | 1 |

[*] BC = British Columbia, AB = Alberta, SK = Saskatchewan, ON = Ontario, QC = Quebec, PE = Prince Edward Island, NS = Nova Scotia, NL = Newfoundland & Labrador, NB = New Brunswick.

# STs not selected for MATS assay (total = 16 STs)

Table Summary

** n= number of isolates

269CC: 168 isolates distributed into 14 STs: ST269 (**n = 142), ST5494 (n = 6), ST13 (n = 3), ST8924 (n = 3); two isolates each of ST479, ST565, ST1986, ST2738; and one isolate each of ST1214, ST11008, ST1161, ST2976, ST8772, ST8880.

41/44 CC: 100 isolates distributed into 45 STs: ST154 (n = 26), ST571 (n = 15), ST2820 (n = 4), ST6617 (n = 4), ST41 (n = 3), ST136 (n = 3); two isolates each of ST44, ST207, ST409, ST5553, ST6169; and one isolate each of ST42, ST110, ST315, ST340, ST1166, ST1194, ST1473, ST1833, ST2288, ST2314, ST5110, ST5550, ST5554, ST6058, ST7612, ST8052, ST8769, ST8771, ST8925, ST9171, ST9352, ST9411, ST9416, ST944, ST10185, ST10187, ST10329, ST10330, ST10324, ST10347, ST10588, ST10619, ST10658, ST11009.

213CC: 14 isolates distributed into 4 STs: ST213 (n = 9), two isolates each of ST3123, ST9413; and one isolate of ST3113.

32CC: 14 isolates distributed into 6 STs: ST32 (n = 8), two isolates of ST6544; and one isolate each of ST290, ST2726, ST7301, ST9410.

35CC: 13 isolates distributed into 9 STs: ST6615 (n = 3), two isolates each of  ST35, ST278; and one isolate each of ST564, ST790, ST3626, ST10657, ST10183, ST10186.

1157CC: 5 isolates of ST1157.

60CC: 4 isolates distributed into 3 STs: two isolates of ST60; and one isolate each of ST11011, ST10587.

162CC: 3 isolates distributed into 2 STs: two isolates of ST162; and one isolate of ST10323.

461CC: 3 isolates of ST461.

865CC: 2 isolates of ST865.

37CC: 1 isolate of ST917.

103CC: 1 isolate of ST4963.

254CC: 1 isolate of ST10332.

Unassigned: 20 isolates distributed into 13 STs: ST5571 (n = 6), ST336 (n = 3); and one isolate each of ST10184, ST10423, ST11018, ST3986, ST7744, ST8894, ST897, ST9248, ST938, ST9409, ST9412.
