# Supplementary material for: Culture-Confirmed Invasive Meningococcal Disease in Canada, 2010 to 2014: Characterization of Serogroup B Neisseria meningitidis Strains and Their Predicted Coverage by the 4CMenB Vaccine
Source: mSphere. 2020 Mar 4;5(2):e00883-19. doi: 10.1128/mSphere.00883-19 (PMC7056808; doi:10.1128/mSphere.00883-19)
Supplement: TABLE S2 [file mSphere.00883-19-st002.pdf]

Supplementary Table S2.

PorA	Number of isolates	Major MenB CCs
VR1 variants		
19-1	126	all CC269
7-2	57	43 of CC41/44
18-7	44	21 of CC41/44 and 23 of CC269
22	32	13 of CC213; 11 Unassigned; 5 of CC41/44
17	13	11 of CC41/44
Others[a]	77	various
VR2 variants		
15-11	126	all CC269
9	48	23 of CC41/44, 24 of CC269
4	40	37 of CC41/44
14	31	13 of CC213, 10 Unassigned
16	15	7 of CC32, three each of CC269 and CC1157
Others[b]	89	various
VR1, VR2 variants		
19-1,15-11	125	all CC 269
18-7,9	43	23 of CC269, 20 of CC41/44
7-2,4	40	37 of CC41/44,
22,14	24	13 of CC213, 9 unassigned
Others[c]	117	various

[a] Other VR1 variants = twelve P1.7; eleven P1.18-1; ten P1.19; seven P1.21; six P1.22-1; five P1.5; four each of P1.12-1, P1.18; three each of P1.5-1, 7-1, 21-7; two each of P1.7-3, P1.31; and one each of P1.12, P1.12-6, P1.17-1, P1.18-3, P1.19-14

[b] Other VR2 variants = nine P1.13-1; eight P1.15; seven P1.14-6; four P1.34; six each of P1.1, P1.2, P1.13, P1.16-3; three each of P1.3, P1.13-2, P1.16-20, P1.25, P1.30-20; two each of P1.16-4, P1.16-5, P1.16-36; and one each of P1.2-2, P1.2-68, P1.9-5, P1.10-46, P1.13-4, P1.13-6, P1.13-9, P1.15-20, P1.16-26, P1.16-66, P1.16-128, P1.23, P1.25-1, P1.30-2, P1.30-22, P1.30-27

[c] Other VR1,VR2 variants = seven each of P1.19,15; P1.22,14-6; six each of P1.7,16; P1.7-2-13; P1.17,16-3; P1.22-1,14; five P1.5,2; four each of P1.12-1,13-1; P1.17,9; P1.18-1,34; three each of P1.7,16-20; P1.7-1,1; P1.7-2,13-2; P1.18,25; P1.18-1,30-20; P1.21,16; P1.21-7,16; two each

of P1.7-2,13-1; P1.7-4,1; P1.17,16-4; P1.18-1,3; P1.19,13-1; P1.21,16-5; P1.21,16-36; and twenty-seven (VR1,VR2) variants that occurred only once (i.e. single isolates).
