# Supplementary material for: Culture-Confirmed Invasive Meningococcal Disease in Canada, 2010 to 2014: Characterization of Serogroup B Neisseria meningitidis Strains and Their Predicted Coverage by the 4CMenB Vaccine
Source: mSphere. 2020 Mar 4;5(2):e00883-19. doi: 10.1128/mSphere.00883-19 (PMC7056808; doi:10.1128/mSphere.00883-19)
Supplement: TABLE S3 [file mSphere.00883-19-st003.pdf]

Supplementary Table S3.

MenB CC	Number Covered/ total (%)	number of isolates covered by antigen* combination fHbp	NHBA	NadA	fHbp + NHBA	NHBA + NadA	PorA + fHbp	PorA + NHBA	PorA + fHbp +NHBA
41/44	62/85 (72.9%)	9	12	0	4	0	6	0	31
269	85/96 (88.5%)	27	4	0	54	0	0	0	0
32	11/13 (84.6%)	5	1	1	3	1	0	0	0
35	7/11 (63.6%)	0	6	0	1	0	0	0	0
37	1/1 (100.0%)	0	1	0	0	0	0	0	0
60	3/3 (100.0%)	3	0	0	0	0	0	0	0
162	3/3 (100.0%)	3	0	0	0	0	0	0	0
213	0/13 (0%)	0	0	0	0	0	0	0	0
461	0/13 (0%)	0	0	0	0	0	0	0	0
865	2/2 (100.0%)	2	0	0	0	0	0	0	0
1157	2/3 (66.7%)	2	0	0	0	0	0	0	0
None assigned	8/18 (44.4%)	6	0	1	0	0	1	0	0

* fHbp = factor H-binding protein; NHBA = *Neisseria* Heparin Binding Antigen; NadA = Neisserial adhesion A; PorA = Class 1 outer membrane protein variable Region 2 antigen P1.4

** None Assigned = Sequence Types not group into any known CC.

Predicted coverage by each antigen: fHbp = (21.6 + 25.2 + 2.8 + 12.4) = 62.8%, 95% confidence interval (46%-65%); NHBA = (9.6 + 25.2 + 0.8 + 0.4 +12.4) = 48.4%, 95% confidence interval (19%-74%); NadA = (0.8 + 0.4) = 1.2%, 95% confidence interval (0.4%-2%), PorA = (2.8 + 0.8 + 12.4) = 16.0%.
