# Supplementary material for: Culture-Confirmed Invasive Meningococcal Disease in Canada, 2010 to 2014: Characterization of Serogroup B Neisseria meningitidis Strains and Their Predicted Coverage by the 4CMenB Vaccine
Source: mSphere. 2020 Mar 4;5(2):e00883-19. doi: 10.1128/mSphere.00883-19 (PMC7056808; doi:10.1128/mSphere.00883-19)
Supplement: TABLE S4 [file mSphere.00883-19-st004.pdf]

Supplementary Table S4.

ST (clonal complex, CC)	Occurring frequency in 250 isolates	Number covered / Number tested (%)	4C MenB vaccine antigen peptides in conferring coverage (Number of isolates covered)			
			fHbp	NHBA	NadA	PorA P1.4
ST269+ (CC269)	74	71/74(95.9%)	15(69), 210(2)	none	none	none
ST154++ (CC41/44)	26	26/26(100.0%)	4(26)	2(25)	none	P1.4(26)
ST213 (CC213)	9	0/9(0.0%)	None	none	none	none
ST571 (CC41/44)	9	3/9(33.3%)	410(1), 687(1)	112(1)	none	none
ST32 (CC32)	7	6/7(85.7%)	1(4), 54(1), 224(1)	none	none	none
ST5571 (none)	6	1/6(16.6%)	631(1)	none	none	none
ST5494 (CC269)	5	4/5(80.0%)	15(3), 506(1)	21(3)	none	none
ST6617 (CC41/44)	4	4/4(100.0%)	509(3),628(1)	none	none	none
6 STs *	3 per ST	10/18(55.6%)	13(2);2,54,332:(1 each)	21(3),10(1)	3(1)	none
19 STs **	2 per ST	25/38(65.8%)	13(5),4(3),15(2);23,108 249,677,688:(1 each)	21(7),2(3),20(2),775(2);3, 771:(1 each)	143(1)	none
54 STs ***	1 per ST	22/54(40.7%)	14(4);4(3);13(2);1,15 256,233,100:(1 each)	2(4),3(2),10(2),21(2); 20, 540,533:(1 each)	1(1)	none

+ Coverage of ST269 isolates were strongly associated with fHbp var 1 peptide 15.

++ Coverage of ST154 isolates were strongly associated with fHbp variant 1 peptide 4, NHBA protein 2 and PorA P1.4.

* STs: ST13, ST136, ST336, ST1157, ST6615, ST8924.

** STs: ST35, ST41, ST44, ST60, ST162, ST207, ST278, ST409, ST461, ST479, ST1986, ST2738, ST2820, ST3123, ST3327, ST6169, ST6554, ST9413, ST9415.

*** STs: ST42, ST110, ST290, ST315, ST340, ST565, ST790, ST897, ST917, ST944, ST1011, ST1161, ST1166, ST1194, ST1214, ST1473, ST1833, ST2288, ST2314, ST2726, ST2976, ST5110, ST5550, ST5553, ST6058, ST7301, ST7612, ST7744, ST8771, ST8894, ST8925, ST9171, ST9248, ST9352, ST9409, ST9411, ST9412, ST9410, ST10183, ST10184, ST10185, ST10186, ST10187, ST10323, ST10329, ST10330, ST10347, ST10423, ST10619, ST10657, ST10658, ST11008, ST11009, ST11018
