# Supplementary material for: Culture-Confirmed Invasive Meningococcal Disease in Canada, 2010 to 2014: Characterization of Serogroup B Neisseria meningitidis Strains and Their Predicted Coverage by the 4CMenB Vaccine
Source: mSphere. 2020 Mar 4;5(2):e00883-19. doi: 10.1128/mSphere.00883-19 (PMC7056808; doi:10.1128/mSphere.00883-19)
Supplement: TABLE S5 [file mSphere.00883-19-st005.pdf]

Supplementary Table S5.

Target	Primer use	Primer (direction)*	Sequence (5' to 3')	Reference**
Orf-2	Serogroup A PCR	98-28(F)	CGCAATAGGTGTATATATTCTTCC	1
		98-29(R)	CGTAATAGTTTCGTATGCCTTCTT	1
siaD	Serogroup B PCR	*siaD* B (F)	CTCTCACCCTCAACCCAATGTC	2
		siaD B (R)	TGTCGGCGGAATAGTAATAATGTT	2
	Serogroup C PCR	*siaD* C (F)	GCACATTCAGGCGGGATTAG	2
		siaD C (R)	TCTCTTGTTGGGCTGTATGGTGTA	2
	Serogroup Y PCR	*siaD* Y (F)	CTCAAAGCGAAGGCTTTGGTTA	3
		siaD Y (R)	CTGAAGCGTTTTCATTATAATTGCTAA	3
	Serogroup W PCR	*siaD* W (F)	CAGAAAGTGAGGGATTTCCATA	3
		siaD W (R)	CACAACCATTTTCATTATAGTTACTGT	3
ctrA	Serogroup E PCR	ctrA 29EF2 (F)	ATTACGCTGACGGCATGTGGA	4
	Serogroup X PCR	ctrA XF3 (F)	GTCTTTGTATAAGGCCCAAG	4
	Serogroup Z PCR	ctrA ZF (F)	TATGCGGTGCTGTTCGCTATG	4
	Serogroup E, X, Z PCR	ctrA UR (R)	TTGTCGCGGATTTGCAACTA	4
porA	PCR and sequencing	P14F (F)	GGGTGTTTGCCCGATGTTTTTAGG	5
	PCR and sequencing	272R (R)	AAGCTGCCAAACAGCCTTCAGCCC	5
	PCR and sequencing	U86F (F)	GCCCTCGTATTGTCCGCACTG	5
	PCR and sequencing	738R (F)	CAGACCGGCATAATACACATCCGA	5
	PCR and sequencing	738F (R)	TCGGATGTGTATTATGCCGGTCTG	5
	PCR and sequencing	P22R (R)	TTAGAATTTGTGGCGCAAACCGAC	5
fHbp	PCR and sequencing	gnal1870F (F)	TGACCTGCCTCATTGATGC	6
	PCR and sequencing	gnal1870R (R)	CGGTAAATTATCGTGTTCGGACGGC	6
	PCR and sequencing	gnal1870v3R (R)	CGTGCCGTCGTGTCCTAG	6
	Sequencing	gnal1870S2 (F)	CAAATCGAAGTGGACGGGCAG	6
	Sequencing	gnal1870S3 (F)	TGTTCGATTTTGCCGTTTCCCTG	6
nhba	PCR and sequencing	gna2132F (F)	GGCGTTCAGACGGCATATTTTTACA	6
	PCR and sequencing	gna2132R (R)	GGTTTATCAACTGATGCGGACTTGA	6
	Sequencing	gna2132S2 (F)	GCGGACACGGTGTCAAAACC	6
	Sequencing	gna2132S4 (F)	GGCGTTCTGCACGGTCGAGG	6
	Sequencing	gna2132S5 (F)	ATGGGTACGCAAAAATTCAA	6
	Sequencing	gna2132S7 (R)	AATGCAGTACTTCGCCGTTGT	6
	Sequencing	gna2132S8 (R)	CCTCGACCGTGCAGAACGCC	6
	Sequencing	gna2132S9 (R)	CCGCACCGCCATTGCCTGTA	6
	Sequencing	gna2132s3new (F)	AGAAAATACAGGCAATGGCGGTGC	6
	Sequencing	gna2132s3s4 (F)	ACGGAAT(G/A)CAGGG(G/T)GACGATCC	6
	Sequencing	gna2132s8s9 (R)	GGATCGTC(A/C)CCCTG(C/T)ATTCCGT	6
nadA	PCR and sequencing	nadAF (F)	GTGGACGTACTCGACTACGAAGG	6
	PCR and sequencing	nadAR (R)	CGAGGCGATTGTCAAACCGTTC	6
	PCR and sequencing	nadAintF (F)	TATGTAAACAAACTTGGTGGGG	6
	PCR and sequencing	nadAintR (R)	GAAATAGAAAAGTTAACAACCAAGTT	6
	Sequencing	nadAS1 (F)	TATGTAAACAAACTTGGTGGGG	6
	Sequencing	nadAS2 (F)	GAAATAGAAAAGTTAACAACCAAGTT	6
	Sequencing	nadAS3 (F)	GACATCAAAGCTGATATCGCTAC	6
	Sequencing	nadAS4 (R)	TTTCGAGGTGGCGCGTTCGGG	6
	Sequencing	nadAS5 (R)	GTAGCGATATCAGCTTTGATGTC	6
	Sequencing	nadAS6 (R)	CTTGGTTGTTAACTTTTCTATTTC	6
	Sequencing	NadA4f (F)	TCAACGGATTCACAGTCGGAGACA	6

Sequencing	NadA4r (R)	CCAATCCATTGGCAGTAGTGTTCAG	6
Sequencing	328-F (F)	GCCATCCTTGCCTCCTTCTG	6
Sequencing	328-R (R)	TTTACCGATAGCAGTCTCGT	6
Sequencing	E030-A (F)	GTCGTGGCTCAACACGACC	6
Sequencing	E030C-R (R)	CCGGACACTTCATCCAGTTTG	6

*F=Forward, R=Reverse

**

1) Muhamed-KT, Simultaneous approach for nonculture PCR-based identification and serogroup prediction of *Neisseria meningitidis*. J Clin Microbiol 2000; 38: 855-857.

2) Pollard AJ, Probe G, Trombley C, et al. Evaluation of a diagnostic polymerase chain reaction assay for *Neisseria meningitidis* in North America and field experience during an outbreak. Arch Pathol Lab Med 2002; 126: 1209-1215.

3) Tzanakaki G, Tsolia M, Vlachou V, et al. Evaluation of non-culture diagnosis of invasive meningococcal disease by polymerase chain reaction (PCR). FEMS Immunol and Med Microbiol 2003; 39: 31-36.

4) Bennett DE, Mulhall RM, Cafferkey MT. PCR-based assay for detection of *Neisseria meningitidis capsular serogroups* 29E, X and Z. J Clin Microbiol 2004; 42 : 1764-1765.

5) Sacchi CT, Lemos APS, Brandt ME, et al. Proposed standardization of *Neisseria meningitidis* PorA variable-region typing nomenclature. Clin and Diagn Lab Immunol 1998; 5 : 845-855.

6) Lucidarme J, Comanducci M, Findlow J, Gray SJ, et al. Characterization of *fhbp*, *nhba* (*gna2132)*, *nadA*, *porA*, and Sequence Type in Group B Meningococcal Case Isolates Collected in England and Wales during January 2008 and Potential Coverage of an Investigational Group B Meningococcal Vaccine. Clin and Vaccine Immunol 2010; 17 : 919-929.
